# Editorial: European partnership on the assessment of risks from chemicals (PARC): focus on new approach methodologies (NAMs) in risk assessment

**DOI:** 10.3389/ftox.2024.1461967

**Published:** 2024-08-08

**Authors:** Louise Ramhøj, Terje Svingen, Tamara Vanhaecke

**Affiliations:** ^1^ National Food Institute, Technical University of Denmark, Lyngby, Denmark; ^2^ Department in vitro Toxicology and Dermato-cosmetology (IVTD), Vrije Universiteit Brussel, Jette, Belgium

**Keywords:** PARC, risk assessment, NGRA, chemical safety, new approach methodologies (NAMs), alternative methods, toxicology

Numerous systems are in place for assessing the potential of chemical substances to cause harm to environmental or human health. However, current risk assessment approaches underlying regulatory decision-making are not adequate for dealing with the ever-increasing number of chemicals and products introduced on the market. Thus, there is an international push to innovate chemical risk assessment through integrated exposure and hazard assessment models and implementing new approach methodologies (NAMs). This next-generation risk assessment (NGRA) paradigm requires integrating and assembling elementary data, with levels of uncertainty, into robust predictions for human risk; an exercise which has proven challenging. To address this challenge, the European Union launched the European Partnership on the Assessment of Risks from Chemicals (PARC) project in 2022 (Marx-Stoelting et al., 2023, and https://www.eu-parc.eu/) to enhance the protection of human health and the environment through a sustainable research and innovation programme.

The PARC project, set to run for 7 years, is divided into several work packages (WPs), with WP5 focusing on hazard assessment for human and environmental health, including more than 80 partners across Europe. WP5 aims to fill data gaps for specified chemical substances of concern (e.g., bisphenol alternatives), improve NGRA approaches as well as adverse outcome pathways (AOPs) and to develop or improve NAMs for chemical hazard assessment. This Research Topic highlights the first activities of a WP5 sub-section specifically relating to the development of NAMs to improve chemical risk assessment for human health. It includes five major research projects that address immunotoxicity, non-genotoxic carcinogenicity, neurotoxicity, and endocrine disruption pertaining to metabolic disorders and the thyroid hormone system.

The first paper in this Research Topic, a minireview by De Castelbajac et al., provides an overview of the objectives and overall structure of the PARC project, with a specific focus on WP5. It introduces the five projects, frames the context of endpoint selection, and discusses how the projects will develop NAMs and their potential regulatory impact.

The paper by Snapkow et al. describes a project aiming to develop NAMs to enhance the risk assessment of immunotoxic chemicals. Traditional methods rely heavily on animal models, such as the T-cell-dependent antibody response (TDAR) and local lymph node assays (LLNA) (Anderson et al., 2011; Lebrec et al., 2014). This project aims to create new *in silico* and *in vitro* tests such as *in vitro* substitutions for the whole blood cytokine release assays (WBCRA), avoiding the need for *ex vivo* material, and an integrated testing strategy for respiratory sensitization potential. Additionally, a review by Hargitai et al. examines the current mechanistic understanding of chemical respiratory sensitization, highlighting knowledge gaps crucial for future NAMs advancements.

The next paper in the Research Topic describes a project dealing with non-genotoxic carcinogens (NGTxCs) (Audebert et al.). Unlike genotoxic substances, which induce DNA damage, NGTxCs cause toxic effects through a broad array of mechanisms, complicating the development of alternative test methods. Long-term animal studies remain necessary but have limited predictivity for human cancer risk. This project aims to develop and improve NAMs for identifying and characterizing NGTxCs and establish a reliable, efficient testing strategy for a safety assessment toolbox.

The third project (Tal et al.) focuses on developing NAMs for developmental (DNT) and adult neurotoxicity (ANT). Chemical exposures can adversely impact the development and function of the nervous system across all stages of life (Grandjean and Landrigan, 2006; Costa et al., 2004). Currently, DNT/ANT data requirements in the EU typically come from *in vivo* studies under OECD test guidelines, with *in vitro* data used for Weight of Evidence assessment for classification and labelling, to trigger further DNT tests, or to support grouping and read-across from similar substances. This project aims to build on an existing DNT *in vitro* test battery that covers several cellular neurodevelopmental processes vital for normal brain development, refining the existing assays, and generating new NAMs to cover essential gaps in the battery, thereby increasing overall cost efficiency and endpoint coverage.

The last two projects aim to develop NAMs to improve the identification of endocrine-disrupting chemicals. The first addresses thyroid hormone system disruptors (Ramhøj et al.). The thyroid hormone signaling axis is a complex network of endocrine regulation, offering numerous entry points for endocrine-disrupting substances to interfere with biomolecules and cause adverse health outcomes (Gilbert et al., 2020). This project focuses on perinatal life as a sensitive window for endocrine disruption and aims to develop a battery of NAMs for human health-relevant risk assessment of thyroid hormone system disruptors. It includes activities to translate or validate assays, leveraging data from zebrafish embryos to inform human health and *in vitro* to *in vivo* extrapolations.

The second endocrine disruptor project focuses on metabolic disorders, specifically developing NAMs to identify endocrine metabolic disruptors (Braeuning et al.). Recognizing that endocrine disruption can occur through mechanisms other than the classic estrogenic, androgenic, thyroid, and steroidogenic (EATS) modalities, this project aims to develop NAMs for non-EATS modalities, such as signaling pathways involved in cellular energy metabolism (Heindel et al., 2017). Disruptions in these pathways are thought to contribute to metabolic disorders like type II diabetes and non-alcoholic fatty liver disease. The project will enhance testing capacity by establishing methods using human-relevant stem cell models, incorporating serum-free conditions and 3D culturing.

With the focus on developing and enhancing NAMs for chemical hazard identification and risk assessment, most of the described projects also link their work to the Adverse Outcome Pathway (AOP) framework (Ankley et al., 2010). The AOP framework organizes existing biological knowledge for humans and wildlife from the perturbation by a stressor leading to an adverse effect in a structured manner, facilitating predictive toxicology capacities. The main repository for AOPs is the AOP-Wiki, a freely available open-access repository supported by the OECD. The last paper in this Research Topic (Jaylet et al.) presents a way to identify well-defined biological areas, as well as gaps within the AOP-Wiki. This approach aids in pinpointing under- and over-represented adverse outcomes to better guide prioritization for further research and development efforts.

This Research Topic of papers highlights ongoing activities within the extensive PARC partnership, specifically focusing on projects aiming to develop NAMs to enhance our capacity to identify harmful chemicals with less reliance on animals for toxicity testing. Importantly, with PARC being a 7-year project, these five projects will be finalized before the end of PARC, but with additional projects being added to the program around halfway through. These will be selected based on identified regulatory needs within the EU.
